# Revision thoracolumbar surgery for flat back deformity: staged ALIF and posterior column osteotomies to avoid three-column osteotomy

**DOI:** 10.3171/2020.1.FocusVid.19740

**Published:** 2020-01-01

**Authors:** Alexandria C. Marino, Thomas J. Buell, Rebecca M. Burke, Tony R. Wang, Chun-Po Yen, Christopher I. Shaffrey, Justin S. Smith

**Affiliations:** 1 Department of Neurological Surgery, University of Virginia Health System, Charlottesville, Virginia; and; 2 Departments of Neurosurgery and Orthopedic Surgery, Duke University Medical Center, Durham, North Carolina

**Keywords:** adult spinal deformity, anterior lumbar interbody fusion, flatback deformity, revision spine surgery, scoliosis, video

## Abstract

Three-column osteotomies (3COs) can achieve significant alignment correction when revising fixed sagittal plane deformities; however, the technique is associated with high complication rates. The authors demonstrate staged anterior-posterior surgery with L5–S1 ALIF (below a prior L3–5 fusion) and multilevel Smith-Petersen osteotomies to circumvent the morbidity associated with 3CO. The patient was a 67-year-old male with three prior lumbar surgeries who presented with back and leg pain. Imaging demonstrated lumbar flat back deformity and sagittal imbalance. The narrated video details key radiological measurements, operative planning and rationale, surgical steps, and outcomes. The patient provided written, informed consent for publication of this illustrative case.

The video can be found here: https://youtu.be/wv4W9D9fUPc.

## Transcript

Three-column osteotomies are useful for revision spine surgery but have high complication rates. To avoid this morbid technique, we used a staged anterior-posterior approach with L5–S1 ALIF and multilevel Smith-Petersen osteotomies for deformity correction.

### 0:42 History and physical

A 67-year-old male presented with back and leg pain. He had three prior lumbar surgeries including a posterior instrumented arthrodesis from L3 to L5. Standing scoliosis films demonstrated global coronal alignment and a thoracolumbar major curve measuring approximately 17°. SVA measured 9 cm. Pelvic tilt was 42°. Pelvic incidence was 71°. Lumbar lordosis was 25°. Thoracic kyphosis was 31°. CT myelogram demonstrated advanced degenerative changes at multiple levels, especially the proximal adjacent levels L1–2, L2–3, as well as T11–12, and the distal adjacent level L5–S1. The patient had high pelvic incidence. We planned to undershoot the PI-LL mismatch and correct lumbar lordosis to approximately 60°. CT scout image is shown at the top right. Given the fixed plane deformity, we could utilize an L4 extended PSO. However, there was a vacuum disc below the prior fusion of reasonable height. We planned to perform a staged anterior-posterior approach with an ALIF at L5–S1 and a posterior revision from T10 to pelvis with Smith-Petersen osteotomies above the prior L3–5 fusion.

### 2:03 Stage 1 ALIF

The patient was positioned supine on a flat-top Jackson table with arms out. An 8-cm left paramedian incision was made between the umbilicus and symphysis pubis. A standard approach to the L5–S1 disc space was taken. Rectus muscles were mobilized and the retroperitoneal space was entered. Fluoroscopy confirmed that the L5–S1 level was exposed. Left common iliac artery and vein are visualized. Middle sacral artery has been ligated between silk sutures and clips. Discectomy was performed and trials were utilized to determine graft size. A 25° hyperlordotic titanium cage was filled with BMP and inserted into the disc space. To facilitate cage stability, a 5 × 22.5–mm screw was placed into the S1 endplate. The cage was still able to be compressed posteriorly during the subsequent stage. Fluoroscopy confirmed adequate placement. Standard closure was performed and initial recovery was uneventful.

### 3:06 Recovery from stage 1

Standing scoliosis films were obtained on postoperative day 3. Global sagittal alignment was 8.5 cm. Pelvic tilt improved from 42° to 30°. Pelvic incidence remained 71°. Lumbar lordosis was improved to 45°. Thoracic kyphosis remained stable at 31°.

We felt we achieved enough correction that the stage 2 posterior revision would utilize Smith-Petersen osteotomies without the need for a pedicle subtraction osteotomy. Details of the posterior revision will be described by my colleague Dr. Marino.

### 3:43 Stage 2 incision and screw placement

For the second stage the patient was positioned prone. An incision was planned. Posterior elements extending from T10 through the ilium were exposed, including previously inserted Steffee plates from L3 to L5, which were removed. Using anatomical landmarks, T10 through S1 as well as iliac screws were placed freehand. Intraoperative CIOS spin imaging confirmed appropriate screw placement without breaches.

### 4:06 Smith-Petersen osteotomies

Smith-Petersen osteotomies were then performed at L1–2 and L2–3. A high-speed drill and osteotome was used to remove the posterior elements at the level of the pars interarticularis en bloc, achieving a Schwab 2 osteotomy.

A Smith-Petersen osteotomy was also completed at L5–S1 at the site of previous decompression.

### 4:44 T11 laminectomy

A T11 laminectomy was performed by drilling lateral troughs that were then completed with Kerrison rongeurs. The lamina was removed. Thickened ligamentum flavum was resected with an Adson rongeur. Adequate decompression was confirmed.

### 5:15 Rod placement

Six-millimeter cobalt chromium rods were cut to span T10 to ilium. We select CoCr rods for potential increased biomechanical construct strength during correction of adult deformities. Gradual rod bending was conducted to correct spinal alignment. With appropriate osteotomies to release the spine, it should bend and conform to its new alignment dictated by the contoured rods. The surgeon bends the rod for lumbar lordosis restoration. The green iliac connector was utilized as we slide the primary rod on the right. Locking caps were placed and tightened with compression applied across the SPO levels to further increase lordosis.

### 6:06 Accessory rod placement

Attention was then turned to accessory rod placement. Side-to-side connectors were placed at T12–L1 and between the S1 and iliac screws to span and reinforce the lumbosacral junction. Accessory rods were cut, bent, and locked in place. Although it would have been preferable to stagger the superior connectors to distribute stress and augment transitional stiffness, this was prevented by the planned tether and the osteotomy.

### 6:42 Tether placement

A tether was then placed. Holes were drilled at the base of the T9 and T8 spinous processes. The tether was passed through the interspinous ligament at T10–11 and then passed through the spinous processes of T9, then through T8, back through T9, and back through the T10–11 interspinous ligament. The tether was secured to the rods and tensioned, being careful to avoid twists in the tether. Excess tether was cut.

### 8:17 Conclusion of operation

Decortication was performed and BMP was placed. Although this is an off-label use, BMP has been shown to promote long-segment fusion in spinal deformity. A mix of autograft and allograft was placed to complete the arthrodesis.

### 8:40 Postoperative course

Global coronal alignment remained adequate. Global sagittal alignment improved to 3.5 cm. Pelvic tilt decreased to 26°. Pelvic incidence remained stable at 71°. Lumbar lordosis increased to 59°, and thoracic kyphosis increased to 41°. Postoperative recovery from stage 2 was uneventful. The patient was ultimately discharged home 7 days after stage 2 with improved back and leg pain. This improvement persisted at his 6-week follow-up visit.
